# Kallikrein 14 activates the chemoattractant protein chemerin in human skin

**DOI:** 10.1016/j.jbc.2025.111002

**Published:** 2025-12-05

**Authors:** Piotr Brzoza, Mariia Tyshchenko, Tomasz Kantyka, Ewa Bielecka, Pawel Majewski, Marcin Migaczewski, Brian A. Zabel, Joanna Cichy

**Affiliations:** 1 Department of Immunology, Faculty of Biochemistry, Biophysics and Biotechnology, Jagiellonian University, Kraków, Poland; 2 Doctoral School of Exact and Natural Sciences, Jagiellonian University, Krakow, Poland; 3 Malopolska Centre of Biotechnology, Jagiellonian University, Kraków, Poland; 4 2nd Department of General Surgery, Jagiellonian University Collegium Medicum, Krakow, Poland; 5 Palo Alto Veterans Institute for Research, VA Palo Alto Health Care System, Palo Alto, California, USA

**Keywords:** chemerin, kallikrein, proteolysis, chemotaxis, skin, oxidation–reduction (redox), immunology, HPLC, mass spectrometr

## Abstract

Chemerin is a multifunctional protein that requires limited carboxyl-terminal proteolysis to manifest its chemotactic, antimicrobial, and metabolic activities. The role of chemerin in the maintenance of skin homeostasis has recently been demonstrated. The main enzymes responsible for processing chemerin in the skin, however, remain elusive. Due to their abundance in the skin and epidermis in particular, kallikreins (KLKs) are promising candidates. Here, using recombinant inactive chemerin, several isolated KLKs, and human primary keratinocytes gene-edited to silence KLK14, we demonstrate that KLK14 is a chemerin-processing enzyme in the skin, rapidly and stably activating this chemoattractant by generating the chemerin 156F and 158S isoforms.

Chemerin is a multifunctional protein best known for its ability to attract various immune cell populations expressing its cognate receptor CMKLR1. These cell populations include plasmacytoid dendritic cells, macrophages, NK cells, and T cells with NK-like activity (1, 2, 3, 4, 5, 6). Moreover, chemerin is involved in a number of metabolic processes, such as adipocyte differentiation (7) and glucose homeostasis (8). Chemerin has also been implicated in angiogenesis (9) and reproductive biology (10). Its involvement in metabolism-related and reproductive processes makes plasma chemerin a potential biomarker for a wide range of diseases. In addition, chemerin and certain chemerin-derived peptides show broad antimicrobial activity, which, together with its presence in epidermis, makes chemerin a player in maintaining skin-barrier homeostasis (11, 12, 13). Changes in cutaneous expression or circulating chemerin levels have been reported in several skin pathologies, most notably psoriasis and atopic dermatitis (14, 15). Recently, chemerin has also been implicated in shaping the tumor microenvironment (3, 16, 17). The main sources of circulating chemerin are thought to be the liver and white adipose tissue (7). Chemerin expression, however, is distributed across many organs, with the Human Protein Atlas database reporting high chemerin mRNA expression levels in endocrine tissues, liver, pancreas, and female reproductive organs. Lower levels are also present in the gastrointestinal tract, lungs, and skeletal muscle as well as the brain (18, 19). Chemerin is synthesized as an inactive precursor (prochemerin) of 163 amino acids, ChemS163, where S163 refers to its C-terminal amino acid. While no chemerin crystal structure has been resolved, the AlphaFold model is available (20), which shows a rigid center protein region with relatively mobile N and C terminus, consistent with its NMR assignment (21). All six cysteine residues are engaged in disulfide bond formation in the native protein, possibly adding to the center region rigidity. This is consistent with the observed chemerin activation mechanism as chemerin requires the removal of several C-terminal amino acids to gain its functions. Chemerin forms that end at serine 157 (ChemS157) were described as being the most active on CMKLR1, resulting in robust chemotaxis of CMKLR1+ cells. Most of the proteases identified to date as capable of processing chemerin are serine and cysteine proteinases involved in the blood coagulation cascade and inflammatory processes, as well as some bacteria-derived enzymes (22, 23, 24). Despite the involvement of chemerin in epidermal homeostasis, there are little data concerning potential ways of chemerin processing by epithelial enzymes. We hypothesize that the kallikrein (KLK) clade of serine proteases comprises likely candidates capable of processing chemerin in the skin. Indeed, KLK7 has already been shown to process chemerin (25). Furthermore, of the 15 members of the KLK family, 11 have been reported in the skin (26) with various spatial expression patterns. Notably, several KLKs, such as KLK5, KLK7, KLK8, KLK13, and KLK14, are located in the outermost epidermal layers (*e.g.*, stratum granulosum and stratum corneum), suggesting that they might control the activity of antimicrobial proteins that are also mostly found in these epidermal layers (27). KLK14 in particular has been described as the most abundant skin-specific KLK, with reported amounts of 240 ng of KLK14 per 1 g of total protein (28). KLKs are known primarily for their role in promoting skin desquamation (29, 30, 31). However, their involvement also appears crucial for proper wound healing, from clot formation, keratinocyte migration, to final wound remodeling (32), processes also linked to chemerin activity (33).

The present study assessed the ability of skin-associated KLKs to activate chemerin, with a major focus on the rapid and stable generation of active chemerin forms at physiological concentrations.

## Results

### KLK14 effectively triggers chemerin activation *in vitro*

**Figure 1:**
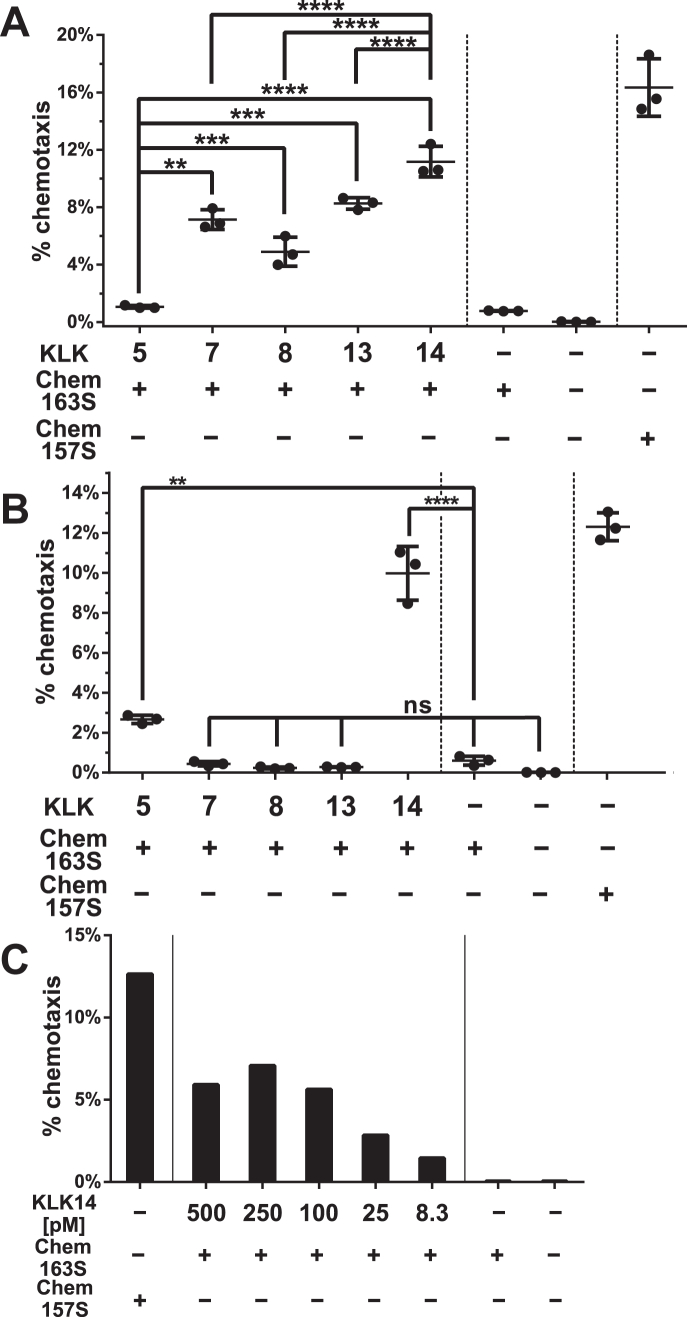
**KLK14 is a potent chemerin activator.** Recombinant human Chem163S was incubated with the indicated KLKs for 2 h at 37 °C. *A,* high concentration (1875 nM:37.5 nM, 50:1 chemerin/KLK molar ratio), (*B*) intermediate concentration (25 nM:250 pM 100:1 chemerin/KLK molar ratio), or (*C*) low/physiologic concentration (25 nM:500–8.3 pM 50:1–3000:1 chemerin/KLK molar ratio). Samples were diluted in chemotaxis media 1875 times (*A*) or 25 times (*B* and C) to obtain equivalent 1 nM concentrations of Chem163S in each sample. Chemotaxis was then assessed using CMKLR1 transfectants by Transwell chemotaxis assay. Cell migration to Chem163S, chemotaxis media only (−), or Chem157S was used as controls. ∗∗*p* < 0.01; ∗∗∗*p* < 0.001; and ∗∗∗∗*p* < 0.0001 by one-way ANOVA with Tukey's post hoc test. Data are shown as the mean ± SD from N = 3 independent experiments (*A* and *B*). Data are from one experiment and are representative of three experiments (*C*). KLK, kallikrein.

To determine the ability of epidermal KLKs to activate chemerin, recombinant human chemerin proform Chem163S was incubated with recombinant human KLK5, KLK7, KLK8, KLK13, or KLK14, followed by a Transwell chemotaxis assay. All KLKs activated chemerin to some degree when applied at high concentrations (Fig. 1*A*). However, at lower and physiologically relevant enzyme concentrations (28), only KLK14 and, to a lesser extent, KLK5, were able to generate active chemerin isoform(s) (Fig. 1*B*). KLK14 cleaved chemerin in a dose-dependent manner and at concentrations as low as 8.3 pM (3000:1, chemerin:KLK14 molar ratio) was able to induce a measurable chemotactic effect, indicating that KLK14 is a potent enzymatic activator of chemerin (Fig. 1*C*).

**Figure 2:**
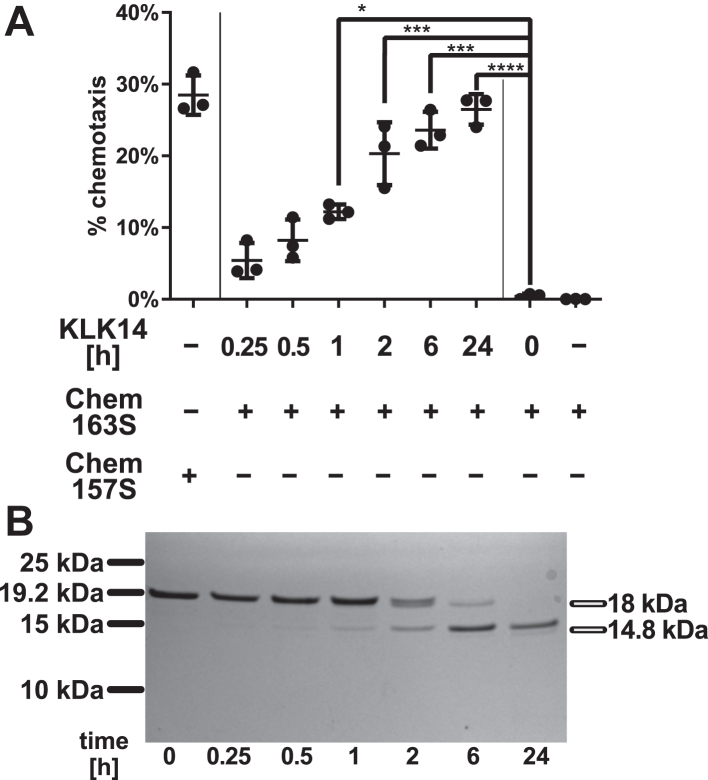
**KLK14 generates stable chemotactic chemerin isoforms in a time-dependent manner.** Recombinant human Chem163S was incubated with the KLK14 at 25 nM:250 pM 100:1 chemerin/KLK molar ratio for the indicated times. *A,* samples were then diluted with chemotaxis media 25 times and assessed by Transwell chemotaxis assay using CMKLR1 transfectants. Cell migration to Chem157S was used as a positive control and to Chem163S as a negative control. ∗*p* < 0.05; ∗∗*p* < 0.01; ∗∗∗*p* < 0.001; and ∗∗∗∗*p* < 0.0001 by one-way ANOVA with Dunnett's post hoc test. Data are shown as the mean ± SD from N = 3 independent experiments. *B,* samples (500 ng per well) were subjected to SDS-PAGE under reducing conditions and visualized using Coomassie blue staining. Data are from one experiment and are representative of three experiments. KLK, kallikrein.

The conversion of Chem163S into active attractant by KLK14 increased over time, and chemerin cleavage products were detected in parallel by SDS-PAGE analysis followed by Coomassie blue protein staining (Fig. 2). The appearance of a strong band at ∼14.8 kDa corresponded with the highest increase in chemotactic activity. At long incubation times (up to 24 h), the ∼14.8 kDa product was the main chemerin fragment visible, indicating a lack of further protein processing or degradation. The functional assay also revealed that prolonged incubation with KLK14 failed to reduce chemotactic activity. Taken together, these data suggest that the resulting active chemerin products are relatively stable and resistant to further proteolysis by KLK14.

### Mapping of KLK14-mediated chemerin cleavage sites and generation of chemotactic fragments

**Figure 3:**
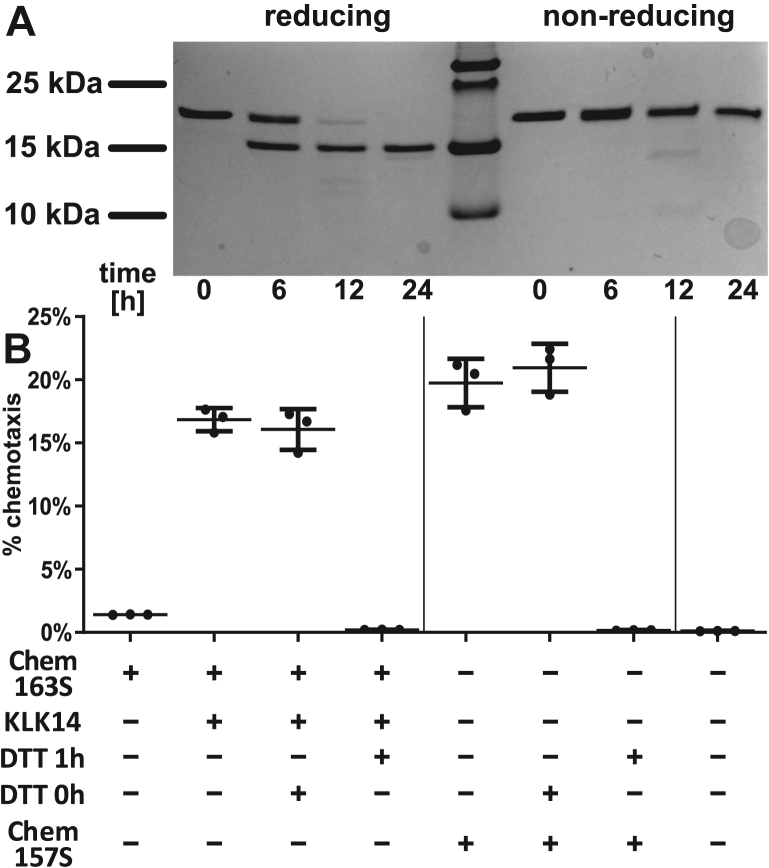
**Disulfide bonds in KLK14-activated chemerin are critical for chemotactic activity.** Chem163S (1875 nM) was treated with KLK14 (37.5 nM) for 2 h at 37 °C. The samples were then subjected to SDS-PAGE under reducing and nonreducing conditions, followed by Coomassie blue staining (*A*) or were further incubated with 5 mM DTT for 60 min at room temperature (DTT 1 h) and subjected to Transwell chemotaxis assay (*B*). As a control for chemotaxis, active Chem157S was treated in a similar way with DTT (5 mM). Samples were then diluted to a final chemerin concentration of 1 nM and 2.7 μM DTT and subjected to a chemotaxis assay. To exclude the direct effect of DTT on cell chemotaxis, 2.7 μM DTT was added to chemotaxis media just prior to chemotaxis (DTT 0 h). Data are shown as the mean ± SD from three experiments. KLK, kallikrein.

Based on the cleavage consensus site of protease KLK14 (28) and the known processing sites of chemerin by other serine proteases, it is likely that KLK14 cleaves Chem163 in the C-terminal domain to release inhibitory residues. The liberation of <10 residues, however, would not be sufficient to account for an apparent loss of 3.2 kDa from Chem163S. Interestingly, reducing and nonreducing gel conditions revealed that the ∼14.8 kDa chemerin cleavage product was apparent only when disulfide bonds were reduced (Fig. 3*A*). This implies that the secondary structure created by disulfide bonds normally keeps chemerin intact despite KLK14-mediated cleavage event(s) that would otherwise release a 3.2 kDa chemerin peptide fragment. To test whether disulfide bonds were essential for observed chemotactic properties, KLK14-cleaved Chem163S was reduced by incubation with DTT for 1 h. Reducing conditions abrogated chemerin chemotactic activity—both for the cleaved Chem163S and the recombinant active Chem157S chemerin isoform, which was used as a positive control (Fig. 3*B*). Addition of DTT to cells just prior to their chemotaxis did not change their chemotactic potential, confirming that DTT used for disulfide bridge reduction of chemerin did not have unintended negative effects on cell migration (Fig. 3*B*). These data suggest that, at least for the tested chemerin isoforms, proper secondary structure is required for effective interaction of chemerin with the CMKLR1 receptor.

### KLK14-derived chemerin cleavage products Chem158K and Chem156F demonstrate chemotactic activity

**Figure 4:**
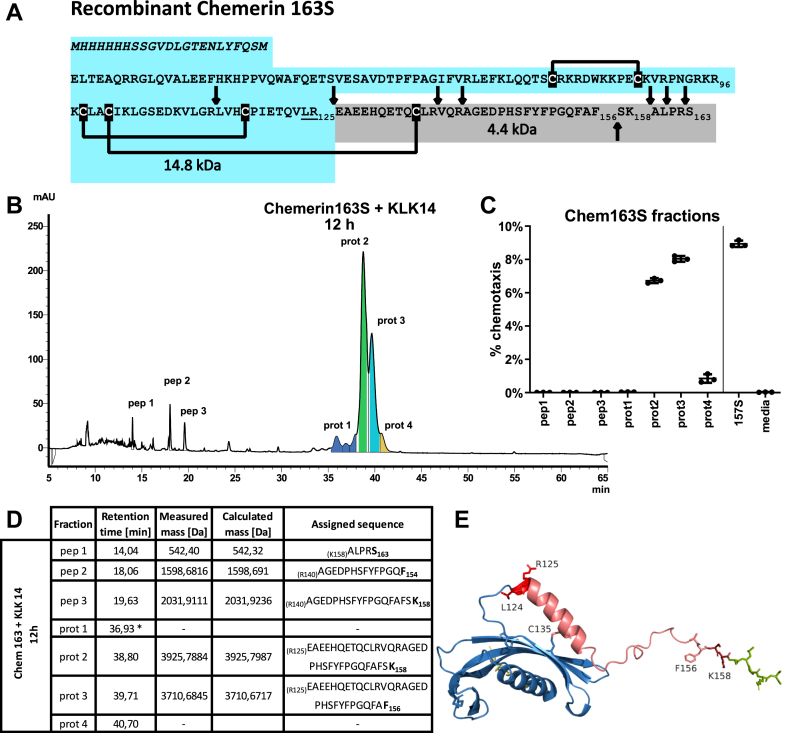
**KLK14 generates chemotactic ChemF156 and ChemK158 isoforms.***A,* recombinant human prochemerin Chem163S sequence with KLK14 cleavage site predictions. Non-native amino acid sequences are in italics, and cysteine bridges are marked. Potential KLK14 cleavage sites are denoted as *arrows*. Proposed products resulting from the R125–E126 KLK14 cleavage, with their masses, are marked. Note that the 4.4 kDa peptide is covalently linked with the bulk of the protein *via* the disulfide bridge through Cys135. *B,* HPLC chromatogram of KLK14-treated Chem163S, resolved in nonreducing conditions, with marked collected peptide and protein fractions. Data are from one experiment and are representative of three experiments. *C,* chemotactic activity of collected fractions. Protein fractions were diluted to 1 nM based on the 280 nm absorbance measurement. Data are shown as the mean ± SD from N = 3 experiments. *D,* collected fractions analyzed using HPLC–MS after iodoacetamide treatment. For protein samples, the liberated C-terminal peptide sequences and masses were identified. *E,* C-terminal domain of prochemerin based on the model generated by AlphaFold (20) with critical residues labeled. KLK, kallikrein.

Prochemerin (Chem163S) has eight potential KLK14 cleavage sites based on conserved sequence motifs (Fig. 4*A*). Interestingly, because of the position of disulfide bonds, while KLK14 cleavage at certain predicted sites would hydrolyze the primary amino acid chain, it would not change the molecular mass or separate peptides from the bulk of the protein. To characterize the cleavage products and chemotactic potential of KLK14-processed chemerin, Chem163S was incubated with KLK14 at 5 μM/100 nM chem:KLK 50:1 molar ratio for 12 h. Chemerin cleavage products were then resolved by HPLC (Fig. 4*B*), and resulting fractions were collected for functional analysis (Fig. 4*C*). Several C-terminal chemerin peptides (pep1–pep3) and larger fragments (prot2 and prot3) were identified by HPLC–MS (Fig. 4*B*). The two major chemerin forms, prot2 and prot3, corresponded to Chem158K and Chem156F, respectively, and displayed strong chemotactic activity (Fig. 4*C*). Other chemerin cleavage products failed to elicit substantial chemotactic activity. In parallel, protein fractions were reduced and treated with iodoacetamide in order to free the C-terminal peptide(s) and reanalyzed by HPLC–MS to determine their sequences (Fig. 4*D*). The KLK14-mediated chemerin cleavage data are in line with the predicted, predominantly trypsin like, and also chymotrypsin-like substrate specificity of KLK14 (Fig. 4*A*). The generation of a ∼14.8 kDa form and the observed band shift in the reducing conditions (Fig. 3*A*) can both be explained by R125–E126 Chem163S cleavage in addition to K158–A159 and F156–S157 hydrolysis. The generated ∼4 kDa protein fragment, ending with F156 and K158, would remain bound to the larger chemerin protein *via* a disulfide bond (Cys101–Cys135), unless reducing conditions were present (Figs. 3*A*, 4, *A*, *D*, and *E*).

**Figure 5:**
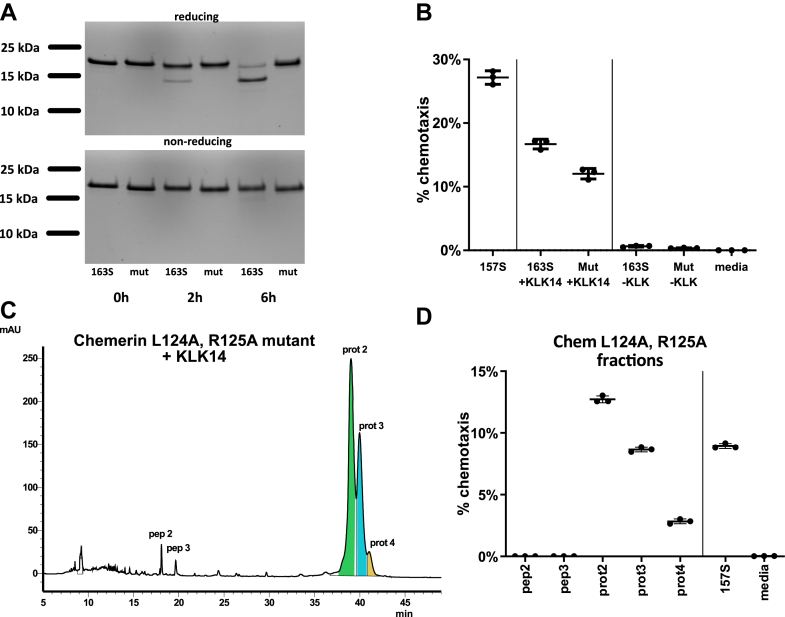
**Chemerin cleavage by KLK14 at position R125–E126 is not critical for chemotactic activation of chemerin.** Chem163S and mutant Chem163S (L124A, R125A, and mut) (1875 nM each) were treated with KLK14 (37.5 nM) for 0, 2, or 6 h at 37 °C. The samples were then subjected to SDS-PAGE under reducing and nonreducing conditions, followed by Coomassie blue staining (*A*). Introduction of the L124A, R125A transitions prevented KLK14 cleavage and release of the ∼4 kDa peptide. The digested samples were diluted to a final chemerin concentration of 1 nM and subjected to a Transwell chemotaxis assay, with Chem157S and media alone as controls (*B*). Mutant chemerin treated with KLK14 retains its chemotactic activity. HPLC analysis of mutant chemerin treated with KLK14 shows a similar peak pattern as native protein Chem163S (*C*). Chemotactic activity of collected fractions. Protein fractions were diluted to 1 nM based on the 280 nm absorbance measurement (*D*). *A* and *C,* data from a single experiment, representative of three experiments. *B* and *D,* data are shown as the mean ± SD from N = 3 experiments. KLK, kallikrein.

**Figure 6:**
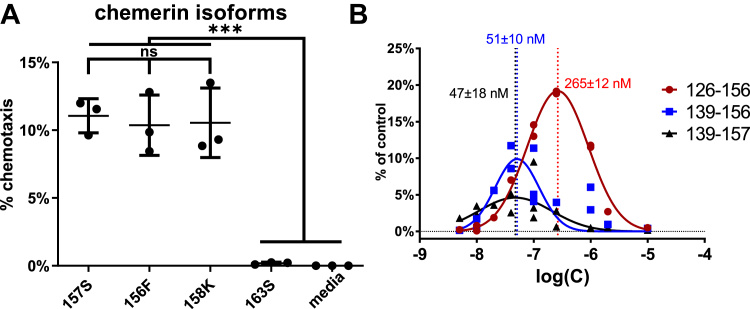
**Chemerin protein isoforms terminated at position F156, S157, and K158 are potent attractants, whereas smaller carboxyl-terminal peptides have weak activity.** Chemerin isoforms 156F, 157S, and 158K show similar chemotactic activity when tested at 1 nM concentrations. Data are shown as the mean ± SD from N = 3 experiments. ∗∗∗*p* < 0.001 by one-way ANOVA with Tukey’s post hoc test (*A*). Chemerin peptides E126–F156, Q139–F156, and Q139–S157 were tested for dose-dependent chemotactic activity by Transwell assay. Data were normalized to the migration of CMKLR1+ cells to 1 nM Chem157S and displayed as “% of control.” The mean concentration of peak cell chemotaxis ± SD is reported for each peptide, N = 3 experiments, with bell-shaped curve fitting by nonlinear regression (*B*).

To determine whether Chem163S cleavage at position R125–E126 is important for chemotactic activity, recombinant mutant chemerin was produced, with the L124 and R125 residues changed to alanines: (LR(124,125)AA). SDS-PAGE under reducing conditions confirmed the lack of generation of the 14.8 kDa cleavage product in the mutant (Fig. 5*A*). By chemotaxis assay, however, mutated chemerin could still be activated by KLK14, indicating that R125–E126 peptide bond proteolysis is dispensable for functional chemerin activation (Fig. 5*B*). HPLC–MS analysis revealed that despite the inability of KLK14 to cleave recombinant mutant chemerin at position A125–E126, the C terminus was modified in a similar way in the mutant as in Chem163S, generating chemotactively active Chem156F and Chem158K isoforms (Fig. 5, *C* and *D*). KLK14-processed native sequence and mutant chemerin have similar HPLC profiles (Figs. 4*B*, 5*C*), even though the E126–F156 or -S157 fragment is bound to the larger chemerin fragment by a disulfide bond (native chemerin) or peptide bond (mutant chemerin). Similar chemotactic activity for both Chem156F and Chem158K was confirmed when, instead of HPLC-purified KLK14 cleavage products, recombinant Chem156F and Chem158K were tested (Fig. 6*A*). We next asked if the E126–F156 peptide in isolation from the rest of the chemerin molecule retained chemotactic activity, as under reducing conditions, it would be expected to be released. E126–F156 was chemically synthesized along with chemerin peptides Q139–F156 and Q139–S157 that were previously reported to be highly active (34). The three peptides exerted relatively weak chemotactic effects (Fig. 6*B*), peaking at ∼20% of that observed for active chemerin protein and requiring concentrations far in excess of the 1 nM active chemerin protein that induces robust cell migration. This indicates that, while the C-terminal peptide sequence is sufficient for weak receptor interactions, secondary chemerin structure is required for maximal activity.

### Characterization of the ability of KLK14 to generate chemoactive chemerin fragments in primary keratinocytes

To determine whether KLK14 can function as a chemerin-activating enzyme in human keratinocytes, we isolated primary keratinocytes from healthy human donors. These cells were transfected to express recombinant chemerin, and gene editing was performed to silence endogenous KLK14. Control samples expressed recombinant chemerin but were not subjected to KLK14 silencing. Keratinocytes were then partially differentiated in 2D cell culture for 72 h using high-calcium culture media, followed by an additional 48 h of culture to collect conditioned media, which were subsequently analyzed in chemotaxis assays.

**Figure 7:**
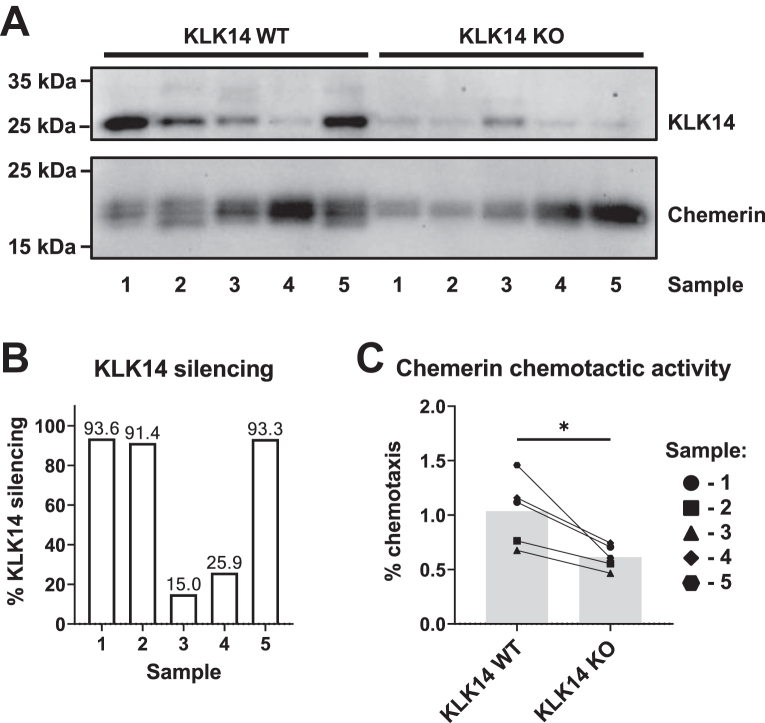
**KLK14 generates chemotactically active chemerin in primary human keratinocytes.** Keratinocytes from five donors were transfected to overexpress chemerin 163S ± gene editing to silence KLK14. Western blotting to assess chemerin and KLK14 levels in keratinocyte culture supernatants (*A*). Quantification of the percent of KLK14 silencing by Western blot densitometry for donor-matched samples (*B*). *C,* chemotactic activity of culture supernatants collected from keratinocytes, such that 1 nM of chemerin was tested for each sample. Data are shown as mean values calculated per donor from two to three experiments; five donors were analyzed across N = 4 independent experiments. ∗*p* < 0.05 by paired *t* test. KLK, kallikrein; KLK KO, chemerin overexpression combined with KLK14 silencing; KLK WT, chemerin overexpression vector alone or combined with silencing control.

Since endogenous chemerin is expressed at very low levels in nonmultilayered keratinocytes (35), this experimental setup allowed us to assess the ability of endogenous KLK14 to generate chemotactically active recombinant chemerin in human keratinocytes. Using keratinocytes from several different donors, with KLK14 silencing levels ranging from 15% to nearly 94% (Fig. 7, *A* and *B*), we demonstrated that in keratinocytes with reduced KLK14 levels, chemerin-dependent chemotaxis was significantly diminished by 38% (Fig. 7*C*).

Together, these data suggest that KLK14 acts as a chemerin-activating enzyme in human keratinocytes.

## Discussion

Here, we describe KLK14 as a potent activator of human chemerin. Prochemerin treated with KLK14 rapidly gains biological activity even at subnanomolar protease concentrations, demonstrating high KLK14 specificity toward prochemerin. Furthermore, the generated active chemerin forms are stable, as even prolonged incubation with KLK14 does not diminish the biological activity of chemerin *via* chemoattractant degradation. This has been verified by both SDS-PAGE and HPLC–MS experiments showing little further fragmentation of chemerin. In contrast, other KLKs tested (with the exception of KLK5, wihch also shows some, albeit markedly lower, activation effects compared with KLK14) require much higher protease concentrations for the biological effect to occur. The chemerin-activating potential of KLK14 was further demonstrated in human keratinocytes, where gene editing–mediated silencing of endogenous KLK14 led to a significantly reduced chemotactic response. These results highlight KLK14 as a chemerin activator in the epidermis.

Although KLK14 protein levels varied notably between keratinocyte donors, multiple studies have shown that KLK14 expression and activity in human skin can differ widely between individuals, even among healthy donors (36, 37, 38, 39). These levels are further modulated by disease state, microbial stimuli (*e.g.*, strong induction of KLK14 following *Staphylococcus aureus* exposure (39), gender (37), and developmental stage, with higher KLK14 expression in fetal *versus* adult skin (40). Together, these data suggest that regulated differences in KLK14 levels may lead to variable capacities of keratinocytes to generate active chemerin.

KLK14-generated chemerin forms were identified by HPLC–MS as Chem156F and Chem158K. Both these isoforms were previously described as products of other chemerin-processing enzymes, like plasmin for Chem158K (24) and KLK7 for Chem156F (25). Generation of the 156F variant alongside the 158K further confirms the dual trypsin-like and chymotrypsin-like substrate specificity of KLK14. The additional R125–E126 cleavage site, while novel, proved to be without consequence for the activation of chemotactic activity, as the active peptide was still covalently attached to the rest of the protein *via* a cysteine bridge likely through Cys101–Cys135. Furthermore, a chemerin mutant incapable of cleavage at R125–E126 was still effectively activated by KLK14 processing.

We hypothesize that the observed additional cleavage at R125–E126 may serve as a rapid way of diminishing the chemotactic activity of KLK14-generated chemerin isoforms. The R125–E126 cleavage site is located at the beginning of the final α-helix based on the chemerin model predicted by AlphaFold, with Cys135 situated in the middle of the helix. In reducing conditions and in the presence of enzymes with reductase activity (like thioredoxin reductase or glutathione reductase), the C-terminal peptide (R125–156F or R125–158K) would be released, leading to dramatically reduced chemotactic activity. Such a mode of action could provide a proteolytic tuning mechanism for the activation/inactivation of chemerin, beneficial for regulating chemerin-dependent inflammation and immune cell homing. A similar type of activity control has been described for the antimicrobial protein psoriasin (41). Furthermore, retaining the C-terminal peptide bound to the rest of the protein may provide a steric shield, preventing further proteolysis by limiting KLK access, which aligns with the observed lack of protein fragmentation/degradation even after prolonged KLK14 digestion. This hypothesis requires the presence of a relatively tightly folded C-terminal region and stands in contrast to the models of prochemerin available (Fig. 4*E*), which present this part of the protein as relatively unstructured and exposed. However, the presence of numerous polar and charged residues within the C terminus suggests possible ionic linkages within the protein, allowing for major conformational changes as already suggested (25). This could be further facilitated by protein relaxation after R125–E126 cleavage. Systematic research focused on structural differences between chemerin isoforms, modes of activation, and chemerin–receptor interactions would provide insight into structure–activity relationships.

In summary, we identify KLK14 as a skin serine protease capable of rapidly and stably generating active chemerin isoforms at subnanomolar concentrations. The role of KLK14 in producing active chemerin in the epidermis is further supported by the reduced chemotactic potential of keratinocyte secretomes when KLK14 is silenced. Identifying the specific sites of KLK14 proteolysis opens the possibility for rapid deactivation of active chemerin isoforms depending on redox conditions. KLK14 and chemerin coexpression in human epidermis (12, 28) and also in deeper skin layers, like dermis (in the case of KLK14 in hair follicles, sebaceous, and sweat glands) (42) suggests that these proteins collaborate to maintain epidermal barrier function and overall skin homeostasis.

## Experimental procedures

### Construction of vectors for expression of different variants of chemerin and for KLK14 silencing

DNA fragments corresponding to the desired chemerin proteins (chemerin coding sequences for Chem157S and Chem163S, lacking the 20-AA N-terminal signal peptide) were amplified by PCR and cloned into the pNIC28-Bsa4 expression vector (Addgene; LGC Standards) using the overlap-extension PCR method (43). pNIC28-Bsa4-chem157 and pNIC28-Bsa4-chem163 constructs were verified by sequencing (Genomed).

Expression vectors for chemerin variants 156F, 158K, and LR(124,125)AA were prepared based on previously obtained vector pNIC28-Bsa4-chem163 using vector mutagenesis. Specific synthetic oligonucleotides were designed to introduce the desired mutation using the overlap-extension method. The identity of the created pNIC28-Bsa4-chem156, pNIC28-Bsa4-chem158, and pNIC-Bsa4-chem124A125A constructs was verified by sequencing (Genomed).

For keratinocyte transfection, a plasmid coding Sleeping Beauty Transposase pCMV(CAT)T7-SB100 (Addgene plasmid #34879; http://n2t.net/addgene:34879; Research Resource Identifier [RRID]: Addgene_34879) (44) was used for integrating the transgene to the mammal genome.

The chemerin overexpression construct was created using the pSBtet-GP vector (Addgene plasmid #60495; http://n2t.net/addgene:60495; RRID: Addgene_60495) (45). Chemerin 163S coding sequence was cloned into the vector by the overlap-extension PCR method, replacing sequence coding for luciferase. Constructs were verified by sequencing.

The expression vector for KLK14 silencing was constructed based on plasmid pSBbi-Pur-dCas9-KRAB-MeCP2_hU6-Sap (Addgene plasmid #196078; http://n2t.net/addgene:196078; RRID: Addgene_196078) (46) by incorporating single-guide RNA targeting KLK14 (CACC ACACTCGGCAGGAGGTCCCG) and hygromycin resistance cassette using the overlap-extension PCR method. The construct was verified by a surveyor assay. As a control, the empty backbone vector (pSBbi-Pur-dCas9-KRAB-MeCP2_hU6-Sap (46)) carrying the hygromycin resistance cassette was used.

### Expression and purification of chemerin variants

All chemerin variants were produced in *Escherichia coli* strain NiCo21(DE3) (New England Biolabs) transformed with plasmids described above and cultured in LB medium. Protein expression was induced by 1 mM IPTG and carried out overnight at 18 °C. Bacteria were harvested, centrifuged, and sonicated in PBS with 1 mM EDTA and cOmplete protease inhibitor (Roche). Sonicated samples were centrifuged (40,000*g*, 20 min, 4 °C), and pellets were resuspended in denaturing buffer (6 M GuHCl, 50 mM NaCl, 50 mM Tris, pH 8) and centrifuged (12,000*g*, 12 min, 4 °C). Remaining supernatants were diluted 100-fold in renaturation buffer (0.5 M GuHCl, 0.4 M sucrose, 0.1 M Tris, 1 mM GSH, 0.1 mM GSSG, pH 9.5). Any precipitate was removed by centrifugation, and the protein solutions were concentrated using Amicon Ultra Centrifugal Filters (Merck). Concentrated solutions were diluted 10-fold in dilution buffer (0.1 M Tris, 1 mM GSH, 0.1 mM GSSG, pH 7.5), and the precipitate was removed. The solution was incubated with Ni-Sepharose 6 Fast Flow (GE Healthcare), and then elution with 500 mM imidazole was conducted. Samples were dialyzed against 25 mM Tris, 1 mM EDTA, 25 mM NaCl, pH 8 (Chem156F 157S and 158K) or 25 mM Tris, 1 mM EDTA, 25 mM NaCl, pH 7.5 (Chem163S and LR(124,125)AA mutant). Finally, protein samples were loaded on Q-Sepharose Fast Flow (GE Healthcare) columns and eluted with increasing concentrations of NaCl. Fractions were collected and analyzed by SDS-PAGE electrophoresis and Coomassie blue staining. Pooled fractions containing proteins of interest were concentrated (Amicon Ultra Centrifugal Filters; Merck), and then dialyzed against PBS. Obtained protein samples were routinely >90% pure as assessed by SDS-PAGE and Coomassie blue staining.

### Expression and purification of KLKs

KLK7, KLK8, KLK13, and KLK14 expression was performed as previously described (47, 48). Briefly, human KLK zymogens were expressed using a *Leishmania tarentolae* system, purified *via* metal affinity chromatography and gel filtration, then activated (either by self-activation or treatment with thermolysin), and titrated (49). Active KLK5 was purchased from R&D Systems.

### Chemerin peptides

Peptides corresponding with the C terminus of chemerin were synthesized by ChinaPeptides (currently QYAOBIO). Supplier confirmed the sequence and >95% purity of the peptides by HPLC–MS. The report is available in the supporting information.

### HPLC, sample collection

Native chemerin or mutated versions were incubated with KLK14 at 7.5 μM Chem:100 nM KLK14 in 75 mM sodium phosphate buffer (pH 7.5) for 12 h at 37 °C. TFA (Sigma) was then added to the final concentration of 0.1%, and samples were briefly centrifuged. Samples were analyzed *via* HPLC. A sample containing 10 μg of chemerin was loaded for each run. Separation was achieved on a reverse-phase column AERIS Widepore XB-C18, 4.6/150 (Phenomenex) connected to Nexera X2 (Shimadzu) UHPLC system. Protein fractions were eluted in the gradient of phase A (0.1% TFA in water) and phase B (80% acetonitrile [ACN], 0.08% TFA in water) and monitored at 215 nm. Collected fractions were vacuum dried and used for further analysis.

### LC–MS analysis

Samples from HPLC fractions were suspended in 25 mM ammonium bicarbonate, reduced with 5 mM DTT for 15 min at 37 °C, and alkylated with 15 mM iodoacetamide for 45 min at room temperature in the dark. Then, ACN was added to the concentration of 2% and TFA to the concentration of about 0.1%. Unfractionated samples of native chemerin and reaction mixture of KLK14-treated chemerin were mixed 1:1 with loading buffer (2% ACN, 0.05% TFA). The LC–MS and LC–MS/MS analyses were performed using micrOTOF-Q II mass spectrometer (Bruker Daltonics) coupled with a nanoHPLC (UltiMate 3000 RSLCnano System; Dionex). Samples were loaded onto a trap column (AcclaimPep-Map100 C18; Thermo Fisher Scientific; ID: 75 μm, length: 20 mm, particle size: 3 μm, and pore size: 100 Å) and then separated on a 15 cm analytical column (AcclaimPepMapRLSC C18; Thermo Fisher Scientific; ID: 75 μm, particle size: 2 μm, and pore size: 100 Å) in a gradient of ACN (2–25% in 2 min, 25–60% in 16 min, 60–90% in 2 min) in the presence of 0.05% formic acid at a flow rate of 300 nl/min. The MS spectra were acquired in a range of 50 to 2500 *m/z*, and the collected data were analyzed using Compass DataAnalysis software, version 4.1 (Bruker Daltonics). In the LC–MS/MS analysis, the measurement cycle consisted of one MS scan and three subsequent MS/MS scans acquired in a data-dependent manner. The data obtained were first processed using Compass DataAnalysis software, version 4.1 (Bruker Daltonics) and then searched *via* the ProteinScape 3.0 platform (Bruker Daltonics) using an in-house MASCOT server (version 2.5.1; Matrix Science) against a common protein contaminant database supplemented with the sequence of chemerin. The following parameters were applied in the search: enzyme—none; variable modifications—oxidation (M), carbamidomethylation (C); peptide mass tolerance—±20 ppm; and fragment mass tolerance—±0.05 Da.

### Chemotactic cell culture

Mouse pre-B lymphoma cell line L1.2 and L.1.2 cells stably transfected with human recombinant CMKLR1 (CMKLR1/L1.2) were cultured in RPMI1640 supplemented with 50 μg/ml gentamicin and 10% fetal bovine serum. The medium for CMKLR1/L1.2 transfectants was also supplemented with 1 mg/ml geneticin.

### Patients and clinical material

All human studies were performed in accordance with the guidelines established by the Jagiellonian University Institutional Bioethics Committee under approved protocols (#87/B/2014; 1072.6120.30.2020) and adhered to the Declaration of Helsinki. Human samples were collected from individuals who were fully informed and had consented. Normal human keratinocytes were isolated from excess skin donated from discarded surgery. Healthy donors included four females and one male, 22, 35, 39 and 40, and 44 years old, respectively.

### Keratinocyte isolation, culture, and modification (chemerin expression, KLK silencing)

Skin tissue samples were washed in calcium- and magnesium-free PBS supplemented with penicillin, 5000 U/ml (Sigma), and streptomycin, 5 mg/ml (Sigma), and digested in dispase solution, 12 U/ml (Gibco), overnight at 4 °C. Then the epidermis was separated using forceps, and keratinocytes were isolated from the epidermis using TrypLE-Select (Gibco). Cells were cultured in KGM-Gold medium (Lonza Group Ltd) at 37 °C with 5% CO_2_. Genetic modifications were performed using FuGene HD Transfection Reagent (Promega) together with proper plasmids in accordance with the producer's instructions. Target protein plasmids were introduced into cells alongside the Sleeping Beauty Transposase vector.

Cells were cultured in KGM-Gold medium until they reached confluency and then differentiated in CnT-Prime 3D Barrier medium (CellNTec) for 72 h. After differentiation, cells were incubated in Dulbecco's modified Eagle's medium (Gibco) supplemented with 1 μg/ml doxycycline (Sigma–Aldrich) for 48 h. Supernatants from differentiated keratinocytes were collected for further analyses.

### Chemotaxis assay

Chem163S was incubated with KLKs in 75 mM sodium phosphate buffer (pH 7.5) (at various concentrations). Alternatively, collected HPLC fractions were used, after resuspension in PBS and measuring protein concentration (bicinchoninic acid and absorbance at λ = 280 nm). For keratinocyte supernatant analysis, chemerin concentration was measured by ELISA according to the manufacturer's recommendations (R&D Systems). Samples were then diluted in cell culture medium to a final concentration of 1 nM chemerin.

Samples were tested in an *in vitro* chemotaxis assay using 5-μm pore Transwell inserts (Costar). A total of 100 μl of cells (2 × 10^5^ cells/well) (input) were added to the top well, and the tested samples were added to the bottom well in a 600 μl volume. Migration was assayed for 2 h at 37 °C. The inserts were then removed, and cells that had migrated through the filter to the lower chamber were collected and counted by flow cytometry (LSRII; BD Biosciences). The results are presented as percent input migration (% chemotaxis).

In each case, enzymatic digestion was stopped by placing samples on ice and diluting to 1 nM chemerin concentration into chemotaxis medium (RPMI1640 containing 10% fetal bovine serum).

### SDS-PAGE and Coomassie staining

Protein samples were mixed with 4x loading buffer (200 mM Tris–HCl [pH 6.8]; 40% glycerol; 8% SDS; 0.4% bromophenol blue with or without 400 mM DTT) and incubated for 20 min at 95°C. The amount of the sample corresponding to 750 ng of undigested chemerin was loaded per lane. Samples were resolved by SDS-PAGE in a 6/16% gradient gel and stained with Coomassie blue.

### Western blot

Supernatant samples from keratinocyte culture were resolved by SDS-PAGE, with 15 μg of total protein loaded per lane (determined by bicinchoninic acid assay), and transferred to polyvinylidene difluoride membranes (Bio-Rad). For chemerin and KLK14 detection, the following antibodies were used: Goat Anti-Human Chemerin Biotinylated Antibody (Abcam; catalog no.: BAF2324), Streptavidin Horseradish Peroxidase (HRP) (BD Pharmingen; catalog no.: BD554066), Rabbit Anti-Human KLK14 (Agrisera; catalog no.: AS06101), and HRP Goat Anti-Rabbit IgG (BD Pharmingen; catalog no.: BD554021). Antibody binding was detected using WesternBright Sirius HRP substrate (Advansta) on ChemiDoc MP imaging system (Bio-Rad) and quantified by densitometry analysis with ImageLab software (Bio-Rad).

### Statistical analysis

Statistical analyses were conducted using GraphPad Prism, version 10 (GraphPad Software, Inc). Data are presented as individual values with mean ± SD marked. Statistical tests used and N values are denoted for each figure. All *p* values below a threshold of 0.05 were considered statistically significant (not significant > 0.05, ∗*p* < 0.05, ∗∗*p* < 0.01, ∗∗∗*p* < 0.001, and ∗∗∗∗*p* < 0.0001).

### Data availability

Experimental data used for this article have been deposited in RODBUK Cracow Open Research Data Repository and are available at https://doi.org/10.57903/UJ/RTQYFX.

## Supporting information

This article contains supporting information.

## Conflict of interest

The authors declare that they have no conflicts of interest with the contents of this article.

## Supporting information

Peptide Certificate
