# Supplementary material for: Kallikrein 14 activates the chemoattractant protein chemerin in human skin
Source: J Biol Chem. 2025 Dec 5;302(1):111002. doi: 10.1016/j.jbc.2025.111002 (PMC12796741; doi:10.1016/j.jbc.2025.111002)
Supplement: Peptide Certificate [file mmc1.pdf]

CERTIFICATE OF ANALYSIS

**Product Name**	Chem[139-156]
**Lot No**	JT-85823
**Sequence**	QRAGEDPHSFYFPGQFAF
**Dissolution condition**	15%ACN+85%H2O
**Length**	18AA
**Modification**	N/A
**Molecular Weight (MW)**	2101.23
**Storage**	-20℃

**Test Items**	**Specifications**	**Results**
**Purity by HPLC**	95%	95.19%
**Peptide Content**	N/A	N/A
**Moisture content**	N/A	N/A
**Acetic acid content**	N/A	N/A
**Appearance**	White to off-white lyophilized powder	Conforms
**Quantity**	1mg	1.0mg

Certified by:
Quality Assurance Department

Date 01-03-2020

Note: this product is intended for research use only; not for diagnostic or human use.

Sample Information

Order ID	: Syn-85823
Name	: Chem[139-156]
Sequence	: QRAGEDPHSFYFPGQFAF
Lot No	: JT-85823
Pump A	: 0.1% Trifluoroacetic in 100% Water
Pump B	: 0.1% Trifluoroacetic in 100% Acetonrtrile
Total Flow	: 1ml/min
Wavelength	: 220nm
Analytial column type	: SHIMADZU Inertsil ODS-SP（4.6*250mm*5um）
Inj. Volume	: 30ul

Time	Module	Action	Value
0.00	Pumps	B.Conc	20
25.00	Pumps	B.Conc	80
25.01	Pumps	B.Conc	100
30.00	Pumps	B.Conc	100
30.01	Pumps	Stop	

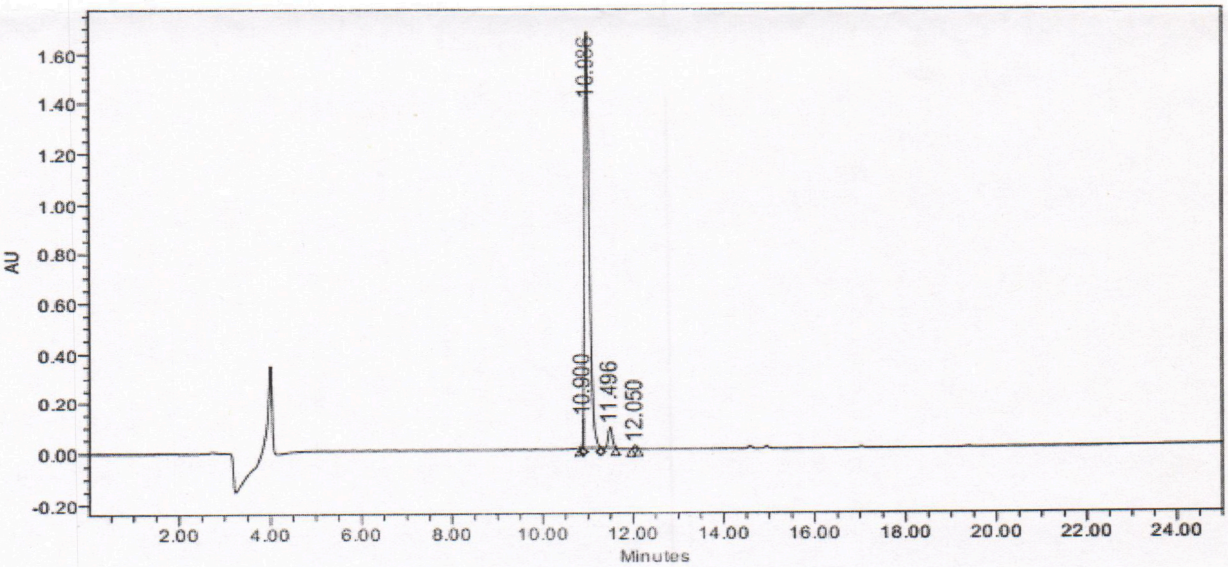

	RT	Area	% Area	Height
1	10.900	88571	0.70	110845
2	10.986	12080357	95.19	1663915
3	11.496	474689	3.74	79419
4	12.050	47558	0.37	9825

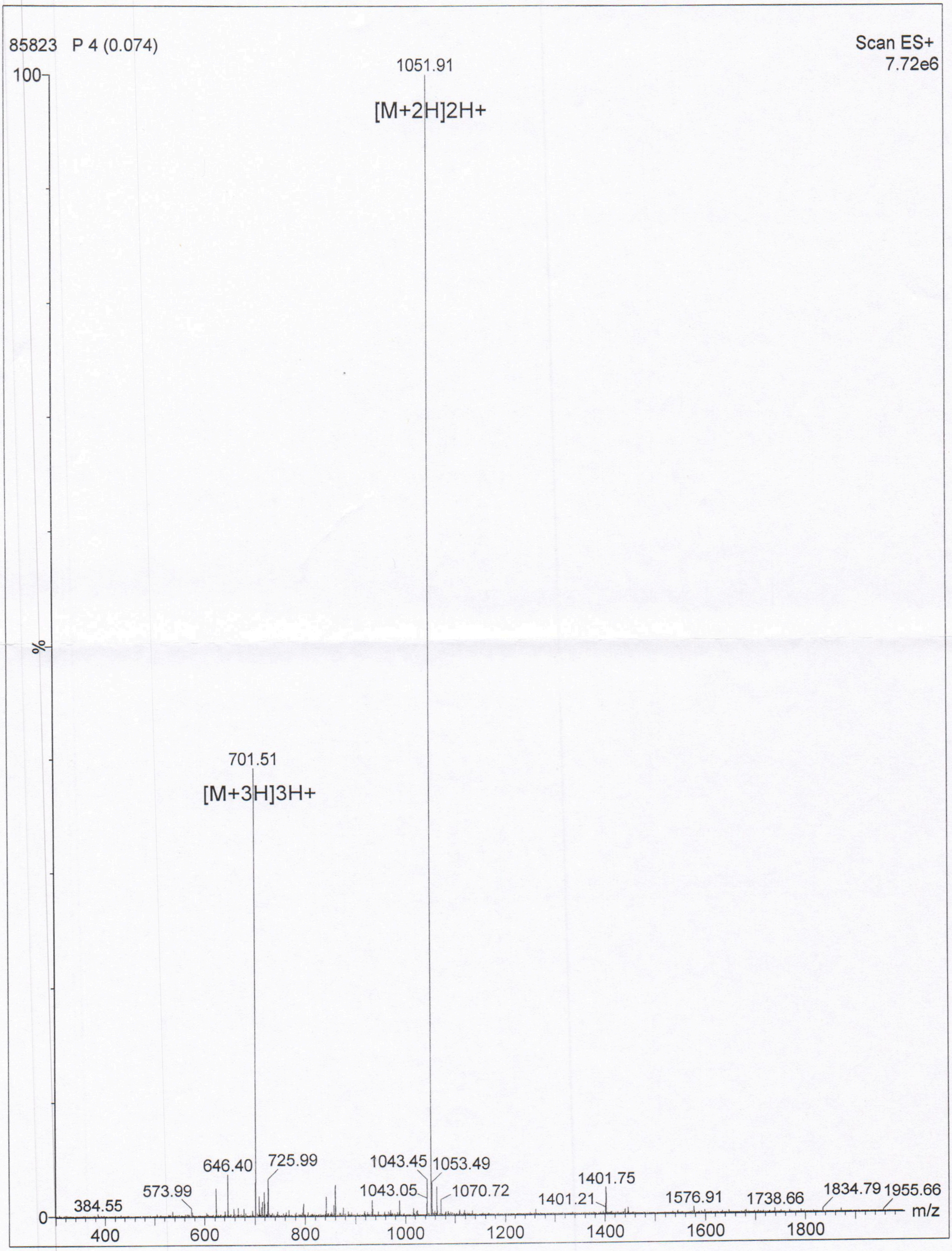
85823 P 4 (0.074)
Scan ES+
7.72e6
1051.91
[M+2H]2H+
701.51
[M+3H]3H+
384.55
573.99
646.40
725.99
1043.45
1053.49
1043.05
1070.72
1401.75
1401.21
1576.91
1738.66
1834.79
1955.66
100
%
0
400
600
800
1000
1200
1400
1600
1800
m/z

Sample Information

Order ID	: Syn-85824
Name	: Chem[139-157]
Sequence	: QRAGEDPHSFYFPGQFAFS
Lot No	: JT-85824
Pump A	: 0.1% Trifluoroacetic in 100% Water
Pump B	: 0.1% Trifluoroacetic in 100% Acetonrtrile
Total Flow	: 1ml/min
Wavelength	: 220nm
Analytial column type	: SHIMADZU Inertsil ODS-SP（4.6*250mm*5um）
Inj. Volume	: 30ul

Time	Module	Action	Value
0.00	Pumps	B.Conc	20
25.00	Pumps	B.Conc	80
25.01	Pumps	B.Conc	100
30.00	Pumps	B.Conc	100
30.01	Pumps	Stop	

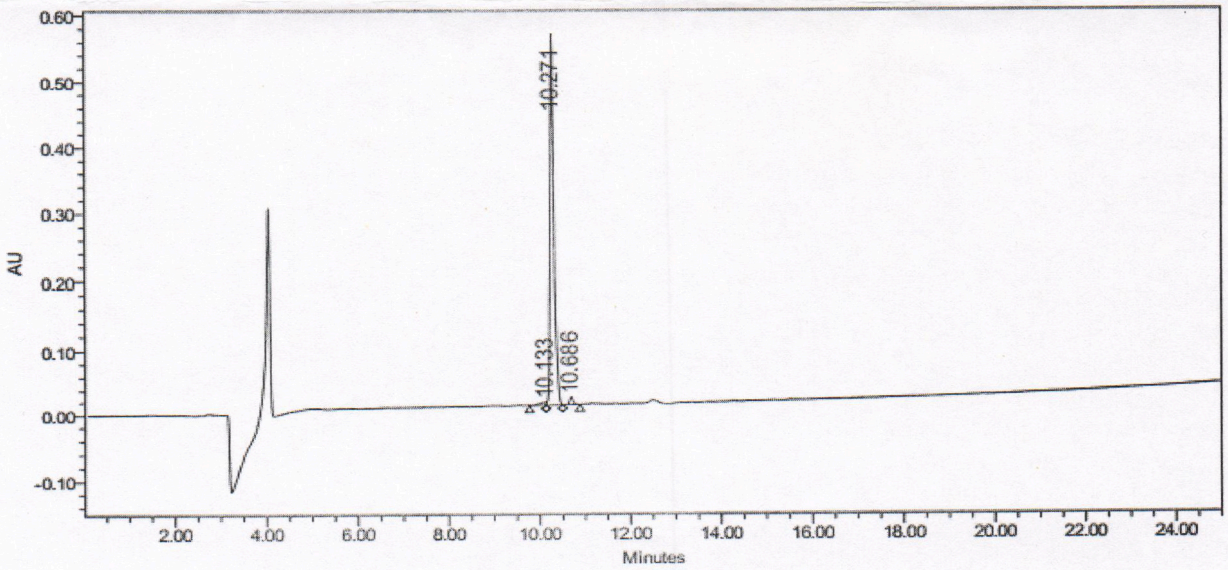

	RT	Area	% Area	Height
1	10.133	42100	1.23	4643
2	10.271	3302404	96.73	553915
3	10.686	69397	2.03	11941

CERTIFICATE OF ANALYSIS

**Product Name**	Chem[139-157]
**Lot No**	JT-85824
**Sequence**	QRAGEDPHSFYFPGQFAFS
**Dissolution condition**	15%ACN+85%H2O
**Length**	19AA
**Modification**	N/A
**Molecular Weight (MW)**	2188.31
**Storage**	-20℃

**Test Items**	**Specifications**	**Results**
**Purity by HPLC**	95%	96.73%
**Peptide Content**	N/A	N/A
**Moisture content**	N/A	N/A
**Acetic acid content**	N/A	N/A
**Appearance**	White to off-white lyophilized powder	Conforms
**Quantity**	1mg	1.0mg

Certified by:
Quality Assurance Department

Date 01-03-2020

Note: this product is intended for research use only; not for diagnostic or human use.

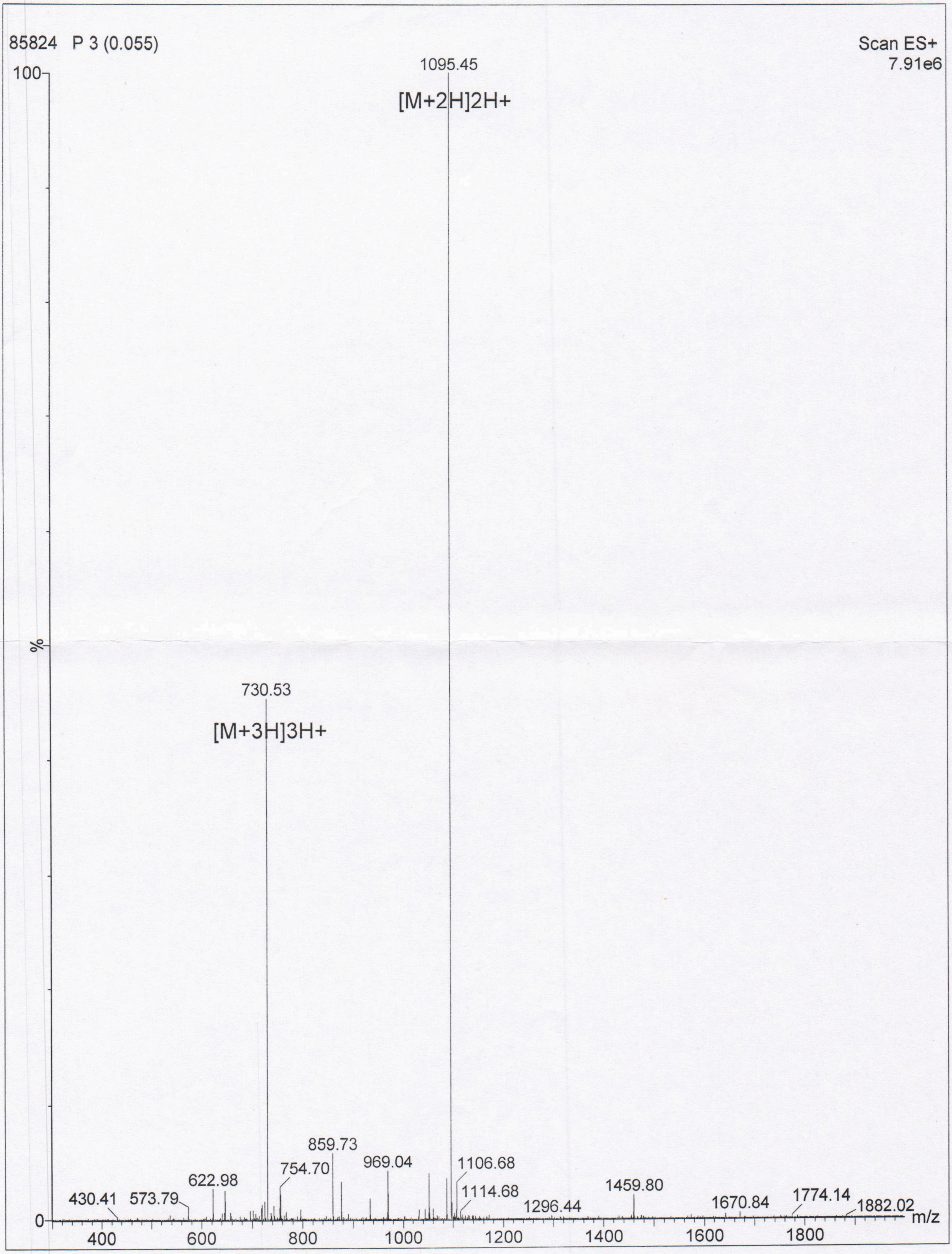
85824 P 3 (0.055)
Scan ES+
7.91e6
100
%
0
1095.45
[M+2H]2H+
730.53
[M+3H]3H+
859.73
754.70
969.04
1106.68
1114.68
622.98
430.41
573.79
1296.44
1459.80
1670.84
1774.14
1882.02
400
600
800
1000
1200
1400
1600
1800
m/z

CERTIFICATE OF ANALYSIS

**Product Name**	Chem[126-156]
**Lot No**	JT-85822
**Sequence**	EAEEHQETQCLRVQRAGEDPHSFYFPGQFAF
**Dissolution condition**	15%ACN+85%H2O
**Length**	31AA
**Modification**	N/A
**Molecular Weight (MW)**	3654.90
**Storage**	-20℃

**Test Items**	**Specifications**	**Results**
**Purity by HPLC**	95%	98.38%
**Peptide Content**	N/A	N/A
**Moisture content**	N/A	N/A
**Acetic acid content**	N/A	N/A
**Appearance**	White to off-white lyophilized powder	Conforms
**Quantity**	1mg	1.0mg

Certified by:
Quality Assurance Department

Date 01-03-2020

Note: this product is intended for research use only; not for diagnostic or human use.

Sample Information

Order ID	: Syn-85822
Name	: Chem[126-156]
Sequence	: EAEEHQETQCLRVQRAGEDPHSFYFPGQFAF
Lot No	: JT-85822
Pump A	: 0.1% Trifluoroacetic in 100% Water
Pump B	: 0.1% Trifluoroacetic in 100% Acetonrtrile
Total Flow	: 1ml/min
Wavelength	: 220nm
Analytial column type	: SHIMADZU Inertsil ODS-SP（4.6*250mm*5um）
Inj. Volume	: 30ul

Time	Module	Action	Value
0.00	Pumps	B.Conc	15
25.00	Pumps	B.Conc	75
25.01	Pumps	B.Conc	100
30.00	Pumps	B.Conc	100
30.01	Pumps	Stop	

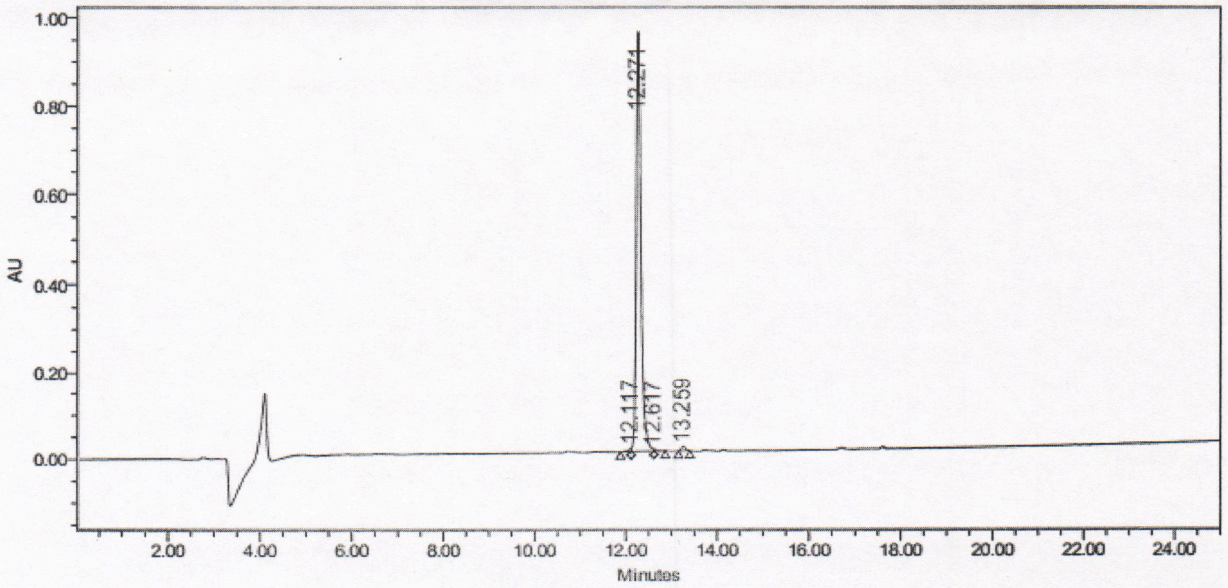

	RT	Area	% Area	Height
1	12.117	35052	0.50	7095
2	12.271	6862188	98.38	951867
3	12.617	15300	0.22	3705
4	13.259	62839	0.90	9628

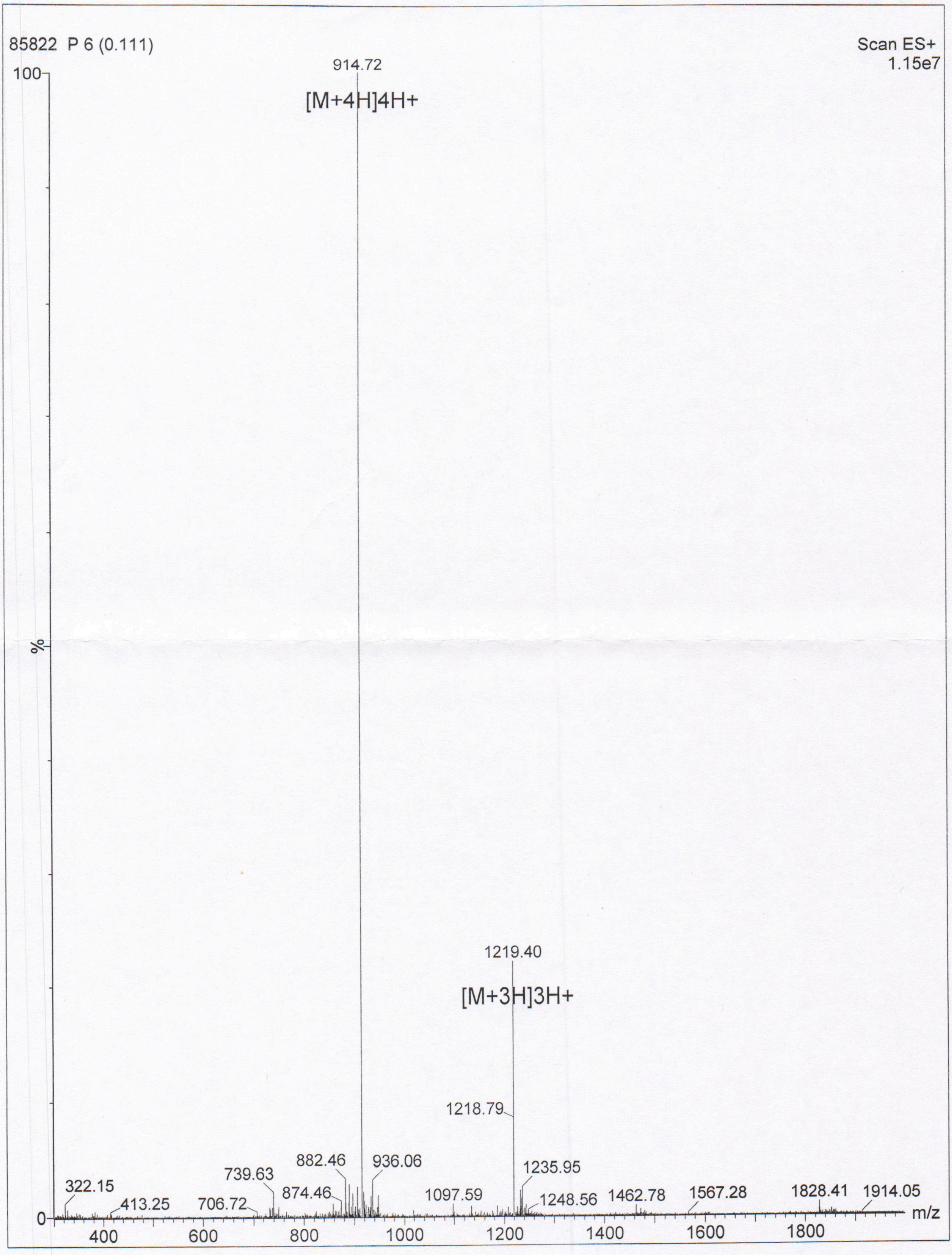
85822 P 6 (0.111)
Scan ES+
1.15e7
914.72
[M+4H]4H+
1219.40
[M+3H]3H+
1218.79
322.15
413.25
706.72
739.63
874.46
882.46
936.06
1097.59
1235.95
1248.56
1462.78
1567.28
1828.41
1914.05
100
%
0
400
600
800
1000
1200
1400
1600
1800
m/z
